# Phase I Clinical Study of the Dietary Supplement, *Agaricus blazei* Murill, in Cancer Patients in Remission

**DOI:** 10.1155/2011/192381

**Published:** 2011-04-18

**Authors:** Satoshi Ohno, Yoshiteru Sumiyoshi, Katsuyoshi Hashine, Akitomi Shirato, Satoru Kyo, Masaki Inoue

**Affiliations:** ^1^Consolidated Research Institute for Advanced Science and Medical Care, Waseda University (ASMeW), 513 Wasedatsurumaki-cho, Shinjuku-ku, Tokyo 162-0041, Japan; ^2^Department of Obstetrics and Gynecology, Graduate School of Medical Science, Kanazawa University, 13-1 Takaramachi, Kanazawa, Ishikawa 920-8640, Japan; ^3^Department of Urology, Gengendo Kisarazu Clinic, 4737 Takayanagi, Kisarazu, Chiba 292-0014, Japan; ^4^Department of Urology, Shikoku Cancer Center, 160 Ko, Minamiumemoto-machi, Matsuyama, Ehime 791-0280, Japan

## Abstract

Although many cancer patients use complementary and alternative medicine, including *Agaricus blazei* Murill (ABM), safety is not yet well understood. Cancer survivors took 1.8, 3.6, or 5.4 g ABM granulated powder (Kyowa Wellness Co., Ltd., Tokyo, Japan) per day orally for 6 months. Adverse events were defined by subjective/objective symptoms and laboratory data according to the National Cancer Institute Common Terminology Criteria for Adverse Events version 3.0 (NCI-CTCAE v3.0). Seventy-eight patients were assessed for safety of ABM (30/24/24 subjects at 1/2/3 packs per day, resp.). Adverse events were observed in 9 patients (12%). Most were digestive in nature such as nausea and diarrhea, and one patient developed a liver dysfunction-related food allergy, drug lymphocyte product. However, none of these adverse events occurred in a dose-dependent manner. This study shows that ABM does not cause problems in most patients within laboratory parameters at the dosages tested over 6 months. This trial supports previous evidence that the ABM product is generally safe, excluding possible allergic reaction.

## 1. Introduction

The prevalence of complementary and alternative medicine (CAM) is increasing worldwide due to growing public interest in natural or holistic therapies and the flow of information via the Internet and mass media. In Japan, the research division supported by a Grant-in-Aid Cancer Research from the Ministry of Health, Labor and Welfare has performed a nationwide cross-sectional survey to evaluate the prevalence of CAM use in cancer patients and their perceptions of cancer CAM, and especially of CAM products used [1]. The survey found a prevalence of CAM use of 44.6% (1382 of 3100) in Japanese cancer patients. The most frequently used CAM products were mushrooms (Agaricus 60.6%, active hexose-correlated compound 8.4%, and *Ganoderma lucidum* 6.3%). 

 Agaricus products are extracted from the *Agaricus blazei* Murill mushroom. *Agaricus blazei* Murill (popularly known as “*Cogumelo do Sol*” in Brazil and “*Himematsutake*” in Japan) is a mushroom native to Brazil and widely cultivated in Japan for medicinal use. In Brazil, Agaricus is traditionally used against a variety of diseases such as diabetes, atherosclerosis, hepatitis, hypercholesterolemia, and heart disease [2]. In Japan, researchers have demonstrated antioxidant, antimutagenic, antitumor, cancer inhibitory, cancer preventive and immunoenhancing effects of *Agaricus blazei* Murill experimentally [3–12]. Experimental studies have especially popularized *Agaricus blazei* Murill mushroom use in Japan, resulting in increased commercial interest stimulating not only production but also registration of new brands and products. No significant toxicity was shown in either the subchronic (SPF-derived F344 rats and beagle dogs) or the two-year chronic bioassay study in experimental animals (SPF-derived F344 rats) (1998~2002) in compliance to the FDA GLP Guideline [13].

Since 1991, *Agaricus blazei* Murill has become a very popular dietary supplement in Japan. For the first 10 years, *Agaricus blazei* Murill consumers had grown to several hundreds of thousands with no reports of any serious side effects associated with *Agaricus blazei* Murill consumption. But in 2001, a clinical case was reported of 3 terminal cancer patients [IIIC ovarian cancer, IIIA, breast cancer, and a metastatic breast cancer (HBV carrier)], who had undergone a regimen of multiple combination chemotherapy and *Agaricus blazei* Murill supplement consumption. All 3 were shown to have severe acute hepatitis with a fatal outcome for 2 and there ensued speculation that the hepatitis could be attributed to *Agaricus blazei* Murill intake [14]. However, throughout the long history of cancer chemotherapy, it has been amply documented (over 1,000 publications) that either a single or a combination chemotherapy either without or with radiation treatment can frequently cause an acute to fatal hepatitis or sudden death due to a lethal potentiation of chemotherapeutic agents by microquantity of endotoxin in cancer patients [15–25]. Furthermore, treatment using radiation or chemotherapeutic agent(s) has been shown to cause a fatal reactivation of chronic hepatitis B virus infection in cancer patients who are chronic hepatitis carriers [26–28]. In contrast, a clinical placebo-controlled toxicity study demonstrated no significant toxicological findings [29, 30]. Numerous other clinical studies with *Agaricus blazei* Murill showed no significant toxicities including acute hepatitis or severe hepatotoxicity [31–41]. Nonetheless, this clinical case report prompted the Japanese Food Safety Committee appointed by Japanese Government to evaluate the safety of *Agaricus blazei* Murill dietary supplement products. In a preliminary genetic toxicity study result, only one of 3 commercial *Agaricus blazei* Murill products tested was reported to be positive. This preliminary Ames' test result was made public on February 13, 2006 by the Ministry of Health, Labor, and Welfare [42]. Immediately after this announcement, the *Agaricus blazei* Murill dietary supplement product shown to be Ames' positive was voluntarily withdrawn from the market with no further production, while all other *Agaricus blazei* Murill dietary supplement producers continued to market their *Agaricus blazei* Murill products. In August, 2006, the *Agaricus blazei* Murill mushroom industry in Japan organized the Agaricus Product Association to establish the guidelines to evaluate the safety, efficacy, quality control and monitoring of all publications relevant to Agaricus dietary supplements [43]. The initial Ames' test results were further scrutinized carefully and more thoroughly by using the transgenic F344 rat LacI, a state-of-the-art genetic toxicity testing system [44]. The outcome demonstrated irrefutable negative results with the identical sample [45], which had been initially announced to be positive and withdrawn from the market. Clearly the initial Ames' test was improperly executed resulting in a false positive [46–49] and too hastily announced by officials without further validation of the preliminary Ames' test results. 

 There are at least 20 manufacturers and approximately 100 Agaricus mushroom-derived dietary supplements. The annual consumption of these dietary supplements derived from *Agaricus blazei* Murill mushroom is approximately 15~20 tons in Japan. Owing to these significant numbers, the Japanese Ministry of Health, Labor, and Welfare has become interested in the safety of commercial Agaricus mushroom supplements among Japanese consumers and therefore supported the objective of this clinical study to evaluate *Agaricus blazei* Murill safety. The rationale for selecting which Agaricus dietary supplements to be tested was based on the annual consumption of each commercial Agaricus dietary supplement. Annually, 70% of Japanese consumers chose Kyowa's Sen-Sei-Ro products (Kyowa Wellness, Co, Ltd., Tokyo, Japan) over those of any other commercial Agaricus products. Unfortunately, due to limited funding for this clinical trial, the study scale had to be restricted to 80 patients. 

 The study product was purchased by public research fund Grant-in-Aid for Cancer Research from the Ministry of Health, Labor and Welfare, Japan. No researcher in this study had any financial or other conflict of interests.

## 2. Subjects and Methods

This study was approved by the institutional review board of the Kanazawa University Hospital and Shikoku Cancer Center. Patients were recruited by the Department of Obstetrics and Gynecology of Kanazawa University Hospital and the Department of Urology of Shikoku Cancer Center, respectively. Prior to enrollment, all patients were provided and required to sign written informed consent. Data management with respect to adverse effects of the test supplement was performed independently by the Monitoring and Evaluation Committee for Clinical Efficacy and Adverse Effects.

### 2.1. Criteria for the Eligibility

Eligibility criteria (Table 1) for inclusion were cancer patients, who were in complete remission with no clinical evidence of relapse of cancer based on (i) a routine follow-up clinical evaluation following radical surgery, radiation, and chemotherapy, (ii) no cancer therapy within 30 days of onset of the study, (iii) no use of dietary supplements, and (iv) no evidence of severe organ dysfunction (aspartate aminotransferase, alanine aminotransferase, alkaline phosphatase, and r-glutamyl transpeptidase levels within twice the upper limits of normal ranges). Patients who met eligibility criteria for the study were recruited between February and August of 2008. 

Demographic and clinical characteristics of the enrolled patients (*n* = 78, 46 male, 32 female) previously treated are shown in Table 1. The mean age of 78 patients was 65.6 ± 10.3 (ages ranged 29~79 years old). Of these 78 patients cancer diagnoses ranged from prostate, endometrial, bladder, ovarian, cervical, gastric, to others that included endometrial stromal sarcoma, uterine sarcoma, vaginal cancer, oral leukoplakia, breast cancer, and renal cell carcinoma of early and advanced stages. Treatment received prior to enrollment included surgery (57 patients), chemotherapy (19 patients), and radiotherapy (30 patients), respectively.

### 2.2. Study Protocols

The chosen test product, “Sen-Sei-Ro Powder Gold, contains 1800 mg lyophilized, granulated powder per pack. One pack contains the following: carbohydrates 820 mg, protein 488 mg, food grade cellulose (dietary fiber) 284 mg, fat 47 mg, and sodium 0.19 mg. According to the 2009 Physician's Desk Reference, water (at 68 mg/pack) contains the following: 0.1 mg Fe, 0.24 mg Ca, 37 mg K, 0.01 mg thiamine, 0.04 mg ergosterol, and 0.59 mg niacin. Results from tests for heavy metals (mercury, cadmium, lead, and arsenic) conformed to strict Japanese food regulations. Safety evaluation was based on laboratory data and subjective/objective symptoms obtained at baseline and after 2, 4, and 6 months of exposure to the test product.

**Figure 1:**
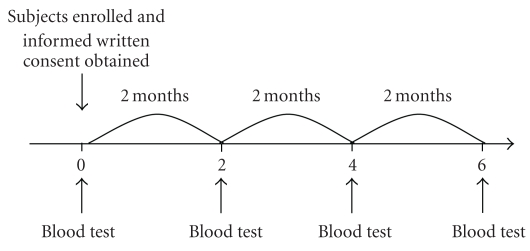
Study schedule.

The first 30 subjects were assigned to the lowest dose group (1 pack/day), the next 24 subjects were assigned to the mid-dose group (2 packs/day), and the last 24 subjects were assigned to the highest dose group (3 packs/day). Subjects diurnally took 1 (1.8 g), 2 (3.6 g), or 3 (5.4 g) packs/day orally for 6 months. The following laboratory parameters were measured: white blood cell (WBC) count, red blood cell (RBC) count, hemoglobin (Hb), hematocrit (Ht), platelet count, aspartate aminotransferase (AST), alanine aminotransferase (ALT), gamma glutamyl transpeptidase (*γ*-GTP), alkaline phosphatase (ALP), total bilirubin (T-bil), total cholesterol (T-cho), total protein (TP), albumin, blood urea nitrogen (BUN), creatinine, sodium (Na), potassium (K), chlorine (Cl), and C-reactive protein (CRP). The intervention schedule is shown in Figure 1.

**Statistical Analysis**

ANOVA analysis was used to identify significant differences in the blood chemistry before and after 1, 2, and 3 packs of Sen-Sei-Ro intake.

### 2.3. Adverse Event Definitions and Causality

Adverse events were defined by subjective/objective symptoms and laboratory data in accordance with the National Cancer Institute Common Terminology Criteria for Adverse Events version 3.0 (NCI-CTCAE v3.0). An adverse event was defined as any undesirable event that occurred to a patient during the course of the study. The definition of a causal relationship between study and adverse event is shown in Table 2.

## 3. Results

### 3.1. Patients

Eighty-one patients met eligibility criteria and enrolled for the study. Of the 81 enrollees, 78 were assessed for safety evaluation of *Agaricus blazei* Murill while three patients withdrew consent for intervention. The three patients who withdrew may have been unable to tolerate the preparation of the finely granulated powder not the Agaricus itself. There were no subjects who could not tolerate the smell and taste of the product. Characteristics of the 78 patients surveyed are shown in Table 1. Compliance was greater than 90% in all patients.

### 3.2. Subjective/Objective Symptoms

A summary of the major adverse events in nine patients (12%) is shown in Table 3. Seven of nine patients discontinued consumption of *Agaricus blazei* Murill granulated powder before the safety trial was completed. In addition, one patient stopped taking the supplement five months after the start of the trial due to diagnosis of a primary tumor recurrence.

 In Table 3, patient no. 55 (59-year-old female, ovarian cancer) was enrolled in the study after cancer-related surgery and six cycles of chemotherapy (Paclitaxel/Carboplatin) as a postoperative adjuvant therapy. Two months after beginning consumption of Agaricus, she complained of generalized urticaria. When she visited the hospital, liver dysfunction (AST 24 (reference value 13–33), ALT 31(ref. 6–27), T-bil 1.7 (ref. 0.3–1.2)), and urticarial papulae were observed. Agaricus consumption was immediately discontinued and she was injected with a hepatoprotector and given oral antiallergy drugs. Three weeks later laboratory data were within normal limits and urticarial papulae had disappeared. A drug lymphocyte stimulation test (DLST) against *Agaricus blazei* Murill product was positive. She had no particular past history of allergic reactions. The causal relationship between *Agaricus blazei* Murill and these adverse events was considered “Definite.”

 Patient no. 19 (69-year-old male, prostate cancer) was enrolled in the study after cancer-related surgery. One month after beginning consumption of Agaricus, he felt nausea. This stopped when he discontinued the supplement but started again when he restarted Agaricus. The causal relationship between Agaricus and this nausea was considered “Probable.” 

Patient no. 4 (47-year-old female) was enrolled in the study one month after a radical hysterectomy for uterine cervical cancer. One month after beginning consumption of Agaricus, she complained of abdominal pain. She discontinued the supplement when diagnostic imaging showed an intestinal obstruction. A week after discontinuation, the obstruction had disappeared. Although she did not restart the supplement, one month later the obstruction reoccurred. The causal relationship between Agaricus and this obstruction was considered “Possible.”

Although transitory digestive symptoms were observed in patients no. 6 (70-year-old male, bladder cancer) and no. 27 (70-years-old male, prostate cancer), they continued in the trial without any treatment for adverse events. The causal relationship between Agaricus and this digestive discomfort was considered “Possible.” Causality of adverse events in other patients (no. 11, no. 32, no. 34, and no. 38) was decided according to the definition shown in Table 2.

### 3.3. Laboratory Data

Adverse events from blood biochemical findings are shown in Table 4. When patients with abnormal levels continued taking the supplement, all laboratory data returned to normal limits without any treatment during the course of study (with the exception of patient no. 55, Table 3). Assessed clinical test items other than those of Table 4 had no apparent abnormalities. No blood chemistry values were significantly different from the pretreatment values with the exception of RBC (*P* = .04), hemoglobin (*P* = .03), hematocrit (*P* = .005), total cholesterol (*P* = .02), TP (*P* = .02), and creatinine (*P* =  .04). However, these differences were mild, transient, and quickly returned to normal (laboratory blood chemistry data not shown).

## 4. Discussion

Despite the numerous reports regarding the preclinical safety and efficacy of *Agaricus blazei* derivatives, further evaluation is needed. 

 It is noteworthy that incidence of acute hepatitis is mostly limited to a single or very few clinical case report(s) among cancer patients, who have undergone chemotherapy with episodic consumption of *Agaricus blazei* Murill, while no such severe acute hepatitis or fatal liver failure was observed in many placebo-controlled clinical studies involving *Agaricus blazei* Murill intake [42–53]. 

**Figure 2:**
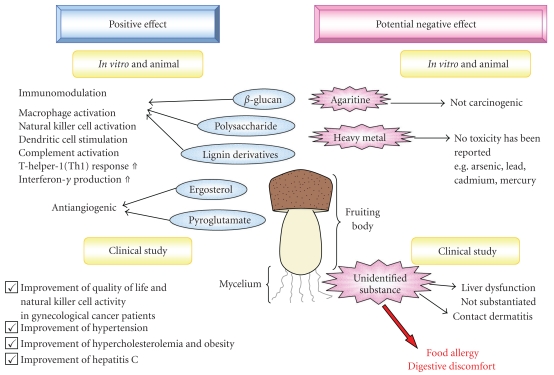
The opposite reaction of *Agaricus blazei* Murill derivatives. Our study of safety clinical trail showed that food allergy and digestive discomfort, associated with *Agaricus blazei* Murill product, were noted as new findings.

Food allergy and digestive discomfort have been recorded as new findings (Figure 2). Additional clinical safety trials are necessary.

Among the 78 subjects, one (1/78 or 1%) food allergy caused by *Agaricus blazei* Murill was observed. Agaricus has been reported to possess biological effects including immunomodulatory and tumoricidal activities, which are due to induction of Th1 response, not the Th2 response induced by IgE-mediated allergies. Moreover, Ellertsen et al. have shown that Agaricus may both prevent allergy development and therapeutically treat established allergies [50]. However, a food allergy to Agaricus is a possible eventuality in all people, including cancer survivors. 

Mushrooms are antigenically rich, but the overall extent of mushroom allergies is unknown [51]. The genus *Agaricus* has been reported as a contact sensitizer in the past [52–55]. Recently, exudative erythema multiforme [56] and chronic cheilitis [57] from *Agaricus blazei* Murill have been reported. Moreover, Herrera-Mozo et al. demonstrated the existence of a new allergen responsible for cross-reactivity between *Agaricus bisporus* and spinach and molds (*Alternaria alternata, Cladosporium herbarum*, and/or *Aspergillus fumigatus*) in allergic patients [58]. At present, the cross-reacting antigen between *Agaricus blazei* Murill and the other foods or allergens has not been identified. The patient (no. 55) who complained of urticaria in this study had nothing particularly noteworthy in her past medical history, including allergic reactions. 

 The present study found that digestive symptoms such as nausea and diarrhea were observed infrequently. In contrast, patients with constipation reported improvement of digestive function, probably as a result of the high dietary fiber (284 mg/pack) contained in *Agaricus blazei* Murill granulated powder. Gastrointestinal manifestations associated with Agaricus may be influenced by patient condition and responsiveness. 

 It is also important to consider natural variability of Agaricus. *Agaricus blazei* Murill dietary supplements are natural products whose chemical compositions vary, depending on factors such as geographic source, climate, and time of harvest of mushroom. Commercially available products also vary from manufacturer to manufacturer in content and concentration of chemical constituents, and even from batch to batch within the same product. Even when natural products are standardized for content of known active or marker compounds to achieve a more consistent pharmaceutical quality, there is nevertheless variation in the concentration of other constituents, which may modulate or potentiate the effectiveness of the active ingredient. These variations can result in differences in pharmacologic activity *in vitro* as well as in bioavailability to humans. Dealing with consistency issues is critical, and in Japan industry groups have recently begun imposing voluntary standards of product safety. Nevertheless, the government may also need to set rules for testing the safety of dietary supplements. 

With regard to safety issues related to consumption of Agaricus mushroom as food or dietary supplements, it contains a wide range of naturally occurring agaritine and heavy metal such as cadmium. The Japanese Food Safety Committee commissioned by the Japanese government was concerned with the potential carcinogenicity of agaritine present in both *Agaricus blazei* and *Agaricus bisporus* despite the existence of several publications of negative results of 2-year chronic carcinogenicity studies with both *Agaricus blazei Murill* [13] *Agaricus bisporus* [59] and the high dose of agaritine [60, 61]. A recent validation study of agaritine using Stratagene's F344 lacI rats failed to demonstrate the presence of DNA adducts of agarithine's potent mutagenic metabolite(s) following daily intake of 80 mg agaritine/kg/daily/90 days [45]. Kyowa's Powder Gold contains 5 ppm of cadmium or less per pack. Hence a regimen of three packs per day equates to a daily cadmium intake of 15 ppm or less. This level of cadmium intake is only 25% of permissible levels in food [62]. 

In conclusion, the present preliminary clinical study (78 subjects) shows that *Agaricus blazei* Murill granulated powder is well tolerated in most patients and that supplement doses of 1.8/3.6/5.4 g per day for 6 months did not cause abnormalities within laboratory parameters. This small-scale clinical trial appears to support the evidence that the *Agaricus blazei* Murill product manufactured by Kyowa Wellness is safe except for the infrequent occurrence of allergic reaction. The present clinical study results provide an important data basis for a larger-scale clinical study with standardized Agaricus product to further validate and support the current findings.

## Figures and Tables

**Table 1:** Inclusion criteria for eligibility and demographic and clinical characteristics of patients previously treated (*n* = 78).

Radical operation for cancer and completely recovered from operation	
No cancer therapy within 30 days	
No use of supplements	
No severe organ dysfunction	
Age 20–80	
Written informed consent	

Number of patients	78
Age (year)	
Mean ± standard deviation	65.6 ± 10.3
Range	29−79
Gender (Male/Female)	46/32

Diagnosis	Number of patients

Prostate cancer (early/advance)	38 (38/0)
Endometrial cancer (early/advance)	12 (8/4)
Bladder cancer (early/advance)	8 (6/2)
Ovarian cancer (early/advance)	6 (3/3)
Cervical cancer (early/advance)	6 (3/3)
Gastric cancer (early/advance)	2 (2/0)
Others (number of patients) (early/advance)	6 (2/4)
Endometrial stromal sarcoma (1), uterine sarcoma (1), vaginal cancer (1), oral cavity cancer (1),	
Breast cancer (1), renal cell carcinoma (1)	

Previous treatment	Number of patients

Operation	57
Chemotherapy	19
Radiotherapy	30

**Table 2:** The definition of a causal relationship between the study and the adverse event.

Variable	Definition
Unrelated	The event is clearly not related to the investigational agent
	*A causal relationship is biologically implausible

Possible	The event may be related to the investigational agent
	*An alternative explanation for the adverse event is present
	For example concurrent administered medication, invasive surgical procedure

Probable	The event is likely to be related to the investigational agent
	*A temporal relationship between the study and the onset of the adverse event is present
	For example recurrence of symptom by readministration
	*An alternative explanation for the adverse event is not present

Definite	The event is clearly related to the investigational agent
	*A causal relationship is biologically explicable

**Table 3:** Summary of the major adverse events.

Patient no.	Sex	Tumor type	Symptoms	Concurrently administered medication	Causality	Safety trial
One pack/day						

Patient no. 6	Male	Bladder cancer	Lower abdominal discomfort (G1)	Betaxolol hydrochloride, nitrendipine	Possible	Complete
Patient no. 11	Male	Prostate cancer	Hand tremor and dizziness when given concomitantly with cold medication (G1)	Cold medication	Possible	Incomplete
Patient no. 19	Male	Prostate cancer	Nausea (G1)	Glimepiride, allopurinol, mecobalamin	Probable	Incomplete
Patient no. 55	Female	Ovarian cancer	Elevated AST (G1) and total bilirubin (G1), urticaria (G3)	—	Definite	Incomplete

Two packs/day						

Patient no. 27	Male	Prostate cancer	Diarrhea (G1)	Allopurinol, losartan potassium	Possible	Complete
Patient no. 32	Male	Prostate cancer	Occurrence of other cancer (G4)		Unrelated	Incomplete
Patient no. 34	Male	Prostate cancer	Nausea (G1)	Nicardipine, trichlormethiazide, carteolol	Possible	Incomplete

Three packs/day						

Patient no. 4	Female	Cervical cancer	Obstruction, gastrointestinal (G2)	—	Possible	Incomplete
Patient no. 38	Male	Prostate cancer	Lower abdominal discomfort (G1)	—	Probable	Incomplete

**Table 4:** Adverse events of blood biochemical findings (*n* = 78).

Event	Daily intake	Grade 1 (%)	≥Grade 2 (%)
Blood/bone marrow			

	1 pack (*n* = 30)	1 (3)	0 (0)
White blood cell	2 packs (*n* = 24)	0 (0)	0 (0)
	3 packs (*n* = 24)	0 (0)	0 (0)

	1 pack (*n* = 30)	2 (7)	0 (0)
Hemoglobin	2 packs (*n* = 24)	2 (8)	0 (0)
	3 packs (*n* = 24)	1 (4)	0 (0)

	1 pack (*n* = 30)	0 (0)	0 (0)
Platelets	2 packs (*n* = 24)	0 (0)	0 (0)
	3 packs (*n* = 24)	0 (0)	0 (0)

Metabolic/laboratory			

	1 pack (*n* = 30)	0 (0)	0 (0)
Aspartate aminotransferase (AST)	2 packs (*n* = 24)	1 (4)	0 (0)
	3 packs (*n* = 24)	2 (8)	0 (0)

	1 pack (*n* = 30)	2 (7)	0 (0)
Alanine aminotransferase (ALT)	2 packs (*n* = 24)	0 (0)	0 (0)
	3 packs (*n* = 24)	0 (0)	0 (0)

	1 pack (*n* = 30)	0 (0)	0 (0)
g-glutamyl transpeptidase (GTP)	2 packs (*n* = 24)	1 (4)	0 (0)
	3 packs (*n* = 24)	0 (0)	0 (0)

	1 pack (*n* = 30)	1 (3)	0 (0)
Alkaline phosphatase	2 packs (*n* = 24)	0 (0)	0 (0)
	3 packs (*n* = 24)	1 (4)	0 (0)

	1 pack (*n* = 30)	3 (10)	0 (0)
Total bilirubin	2 packs (*n* = 24)	0 (0)	0 (0)
	3 packs (*n* = 24)	0 (0)	0 (0)

	1 pack (*n* = 30)	0 (0)	0 (0)
Total cholesterol	2 packs (*n* = 24)	1 (4)	0 (0)
	3 packs (*n* = 24)	0 (0)	0 (0)

	1 pack (*n* = 30)	2 (7)	0 (0)
Albumin	2 packs (*n* = 24)	0 (0)	0 (0)
	3 packs (*n* = 24)	0 (0)	0 (0)

	1 pack (*n* = 30)	0 (0)	0 (0)
Creatinine	2 packs (*n* = 24)	0 (0)	0 (0)
	3 packs (*n* = 24)	1 (4)	0 (0)

	1 pack (*n* = 30)	1 (3)	0 (0)
Na, high	2 packs (*n* = 24)	0 (0)	0 (0)
	3 packs (*n* = 24)	0 (0)	0 (0)

	1 pack (*n* = 30)	2 (7)	0 (0)
K, high	2 packs (*n* = 24)	0 (0)	0 (0)
	3 packs (*n* = 24)	1 (4)	0 (0)

	1 pack (*n* = 30)	1 (4)	0 (0)
K, low	2 packs (*n* = 24)	0 (0)	0 (0)
	3 packs (*n* = 24)	0 (0)	0 (0)
